# A new species of Middle Miocene baleen whale from the Nupinai Group, Hikatagawa Formation of Hokkaido, Japan

**DOI:** 10.7717/peerj.4934

**Published:** 2018-06-26

**Authors:** Yoshihiro Tanaka, Tatsuro Ando, Hiroshi Sawamura

**Affiliations:** 1 Osaka Museum of Natural History, Osaka, Japan; 2 Hokkaido University Museum, Sapporo, Japan; 3 Numata Fossil Museum, Hokkaido, Japan; 4 Ashoro Museum of Paleontology, Hokkaido, Japan

**Keywords:** Fossil, Baleen whale, New genus, New species, Mysticeti

## Abstract

A fossil whale from the Hikatagawa Formation (Middle Miocene, 15.2–11.5 Ma) of Hokkaido, Japan is described as a new genus and species *Taikicetus inouei* and its phylogenetic position is examined. Consistent with the result of Marx, Lambert & de Muizon (2017), the Cetotheriidae form a clade with the Balaenopteroidea, and “a clade comprising *Isanacetus, Parietobalaena* and related taxa” is located basal to the Balaenopteroidea + Cetotheriidae clade. *Taikicetus inouei* is placed in the clade with most of members of “Cetotheres” sensu lato comprising *Isanacetus, Parietobalaena* and related taxa. *Taikicetus inouei* can be distinguished from the other members of “Cetotheres” sensu lato in having an anteriorly swollen short zygomatic process, high triangular coronoid process, and angular process, which does not reach as far posterior as the mandibular condyle. *Taikicetus inouei* is only record of “Cetotheres” sensu lato from Hokkaido, Japan and the northern-most records of “Cetotheres” sensu lato in Japan.

## Introduction

The systematics of the Cetotheriidae have been controversial. The family Cetotheriidae was originally established by Brandt (1872) with limited use until Miller (1923) stated the context of Cetotheriidae as “*Cetotherium* and related genera” without defining the contents. Later, Simpson (1945) and later authors treated the Cetotheriidae as a much larger group.

Several authors expressed concerns about the broadly defined family Cetotheriidae. Kellogg (1928) said the internal relationships of the Cetotheriidae are “incompletely known”. Fordyce & de Muizon (2001) stated that the Cetotheriidae are paraphyletic. Despite such concerns, Cetotheriidae have continued to play a role as a “wastebasket taxon”, as mentioned by Marx, Bosselaers & Louwye (2016).

Bouetel & de Muizon (2006) used the term Cetotheriidae sensu stricto and listed the members of the Cetotheriidae sensu stricto explicitly. Deméré et al. (2008) confirmed the monophyletic Cetotheriidae sensu stricto and recognized a monophyletic group including other so-called cetotheriids that form a clade with the Cetotheriidae sensu stricto. Kimura & Hasegawa (2010) called the traditional Cetotheriide as Cetotheriidae sensu lato and divided it into the Cetotheriidae sensu stricto and *Isanacetus*-group, which was previously established by Kimura & Ozawa (2002). They also found that the *Isanacetus*-group was monophyletic. Bisconti, Lambert & Bosselaers (2013) identified other cetotheriids paraphyletic and characterized them as “basal thalassotherians the thalassotherian stem group or Thalassotherii *sedis mutabilis*”.

Boessenecker & Fordyce (2015) and Marx & Fordyce (2015) comprehensively recognized the clade too and Boessenecker & Fordyce (2015) called the clade “Cetotheres” sensu lato. Marx & Fordyce (2015) implied that some groupings with the clade “may indicate the existence of previously unrecognized family-level taxa”. Marx, Lambert & de Muizon (2017) defined the “Cetotheres” sensu lato as a group including *Aglaocetus, Cophocetus, Diorocetus, Isanacetus, Parietobalaena, Pelocetus, Thinocetus* and *Uranocetus*, thus excluding *Thinocetus, Pelocetus* and *Uranocetus*. In spite of this, Marx, Lambert & de Muizon (2017) considered that the clade was poorly defined stating that “none of which appear to share unequivocal similarities with either each other or any of the established families”. Marx, Lambert & de Muizon (2017) considered that “a clade comprising *Isanacetus, Parietobalaena* and related taxa” were within the “Cetotheres” sensu lato.

Here, we use the terms of Cetotheriidae sensu stricto sensu Bouetel & de Muizon (2006), and the clade including previously so-called cetotheriids, which are currently excluded from Cetotheriidae sensu stricto as the “Cetotheres” sensu lato consistent with Marx, Lambert & de Muizon (2017).

**Figure 1:**
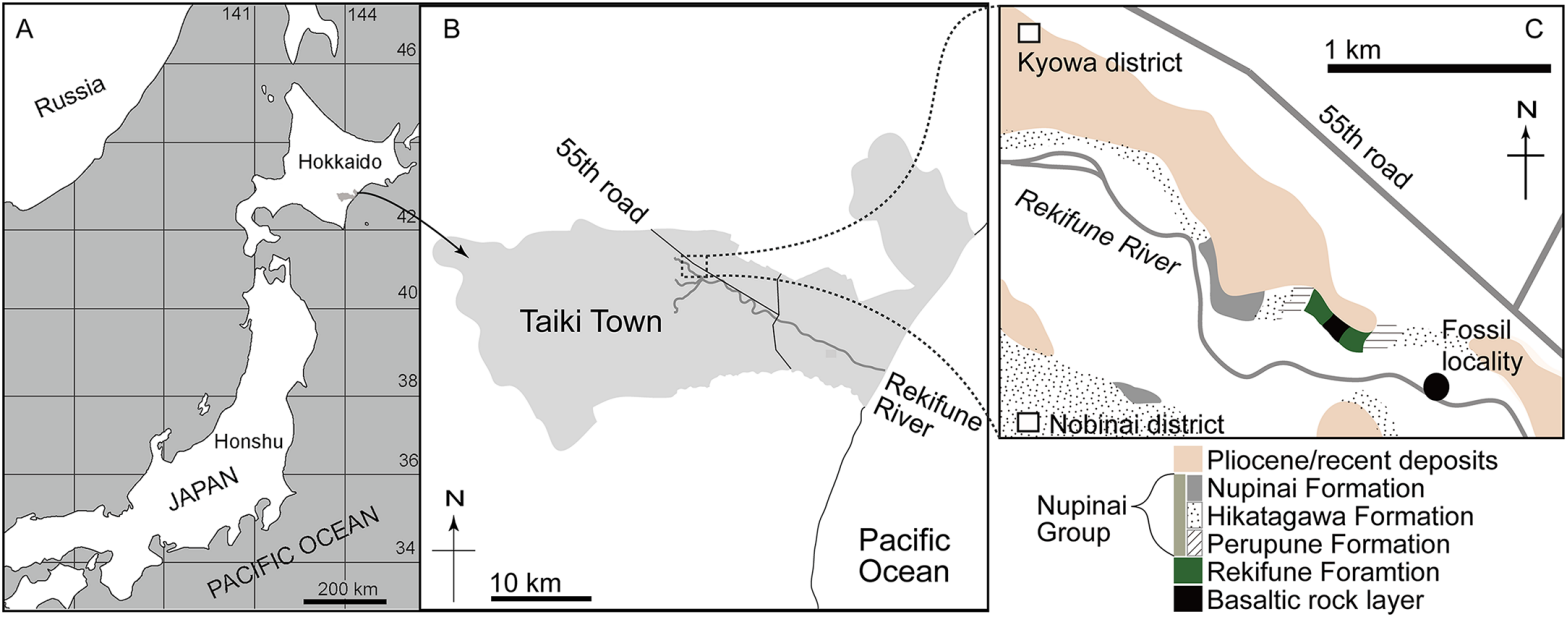
Maps showing the locality of AMP 35, *Taikicetus inouei* at Taiki Town, Hokkaido, Japan, reported in this study. (A) Larger map, modified from Tanaka & Kohno (2015); (B) local map; (C) local map showing the type locality.

A mysticete fossil from the Middle Miocene of Hokkaido, Japan (Fig. 1), AMP 35 was originally reported by Egashira & Kimura (1998). They described AMP 35 as “the first cetotheriid from Hokkaido”, Cetotheriidae gen. et sp. indet. based on two features from Miller (1923): (1) having a gradually downward and outward sloped supraorbital process of the frontal and (2) narrow nasals, which are equal to less than half of the length of the supraorbital portion of the frontal. They listed an additional nine features to identify AMP 35 as Cetotheriidae, but those features were shared with the Balaenopteridae and/or Eschrichtiidae (see Table 4 of Egashira & Kimura, 1998). As stated above, the definition of the Cetotheriidae has been changed and those features are neither adequate nor appropriate to diagnose Cetotheriidae. Tanaka, Furusawa & Barnes (in press) provisionally suggested that AMP 35 was not a member of the Cetotheriidae sensu stricto, based on its weak angular process, which does not reach as far posterior as the mandibular condyle. Because a long angular process of the mandible is a synapomorphy of the Cetotheriidae sensu stricto (El Adli, Deméré & Boessenecker, 2014). AMP 35 is identified here as a new genus and species, and our phylogenetic analysis places it in an unnamed clade of “a clade comprising *Isanacetus, Parietobalaena* and related taxa”. This clade includes most species of “Cetotheres” sensu lato.

## Materials and Methods

**Material:** AMP 35, a right posterior part of the skull (premaxillae, maxillae, frontals, nasals, vomer, palatines, parietals, pterygoid, basisphenoid/presphenoid, basioccipital, supraoccipital, exoccipital, squamosal), right periotic in situ, right tympanic, mandible and atlas. Morphological terms follow Mead & Fordyce (2009) for the skull and earbones. All the figures show the specimen coated with sublimed ammonium chloride.

**Nomenclatural acts:** The electronic version of this article in Portable Document Format will represent a published work according to the International Commission on Zoological Nomenclature (ICZN), and hence the new names contained in the electronic version are effectively published under that Code from the electronic edition alone. This published work and the nomenclatural acts it contains have been registered in ZooBank, the online registration system for the ICZN. The ZooBank Life Science Identifiers (LSIDs) can be resolved and the associated information viewed through any standard web browser by appending the LSID to the prefix http://zoobank.org/. The LSID for this publication is: urn:lsid:zoobank.org:pub:C2190D5F-D3FF-482F-8580-DF8057B2F3AF. The online version of this work is archived and available from the following digital repositories: PeerJ, PubMed Central and CLOCKSS.

**Phylogenetic analysis:** The phylogenetic position of AMP 35 was analyzed using the morphological partition of the matrix of Marx, Lambert & de Muizon (2017), which contains 272 morphological characters and 95 taxa, with additions of AMP 35. The data matrix in nexus and TNT formats, the character list, and the tree file are in the Data S1–S4 respectively. Percentages of coded data of AMP 35 are 25% (includes soft tissue characters) and 26% (excludes soft tissue), 20% for the periotic and 8% for the tympanic bulla.

Character data and tree data were managed using Mesquite 2.75 (Maddison & Maddison, 2011). Analysis was performed with TNT, 1.5 (Goloboff & Catalano, 2016). All of characters were treated as unweighted. Different from Marx, Lambert & de Muizon (2017) all characters were considered unordered to avoid a priori assumption on the polarity of the character states, and with a backbone constraint of extant taxa, based on the topology of the molecular phylogeny of Steeman et al. (2009). The analysis used the New Technology Search with find minimum length trees = 1,000 times.

## Results

### Systematic paleontology

Cetacea Brisson, 1762
Neoceti Fordyce & de Muizon, 2001
Mysticeti Gray, 1864
Taikicetus, gen. nov.

**Type and only included species:** *Taikicetus inouei*, new species.

**Diagnosis:** As for the only included species, *Taikicetus inouei*, below.

**Etymology:** Named after the locality of the type specimen, Taiki Town, Hokkaido, Japan. Cetus or cete is whale in Latin.

Taikicetus inouei sp. nov.
(Figs. 2–10 and Tables 1–3)

**Table 1:** Measurements in millimeter of skull, AMP 35, *Taikicetus inouei* following Boessenecker (2013).

	AMP 35
Total length as preserved	435
Breadth across exoccipitals	238*
Breadth across postglenoid processes	262*
Zygomatic width	280*
Length of temporal fossa	180
Transverse width of temporal fossa	262*
Posterior cranium to preorbital process	229+
Transverse width across basioccipital crests	95*
Width of supraoccipital at vertex	24
Length of supraoccipital	256

**Note:**

Measurements are rounded to the nearest 0.5 mm. The asterisk (*) means only one side.

**Table 2:** Measurements in millimeter of mandible, AMP 35, *Taikicetus inouei* following Tsai & Fordyce (2015).

Mandibles	AMP 35
Length of mandible, as preserved	460.0
Height of mandible, from coronoid process of ventral margin	142.0
Maximum preserved height of mandible	142.0
Maximum preserved width of mandible	63.0

**Note:**

Measurements are rounded to the nearest 0.5 mm.

**Table 3:** Measurements in millimeter of periotics and bulla, AMP 35, *Taikicetus inouei* following Tsai & Fordyce (2015).

	AMP 35
**Periotic**	
Greatest length of aperture for the cochlear aqueduct	118
Length of anterior process, from anteroventral angle to anterior incisure	30
**Bulla**	
Greatest length, in lateral view	85.5
Greatest width, in latera view	45.5
Greatest height, from tip of sigmoid process	47.0+
Length from tip of sigmoid process to anterior tip of tympanic bulla (from tip to tip)	53.0+
Length from tip of sigmoid process to anterior tip of tympanic bulla (from cross-section to tip)	42.5+

**Note:**

Measurements are rounded to the nearest 0.5 mm.

**Etymology:** Inouei is named after Mr. Kiyokazu Inoue who found the type specimen. The name, *Taikicetus inouei* was formerly suggested by Professor Masaichi Kimura and Mr. Fumio Egashira at a press conference in Feb. 1995.

**Diagnosis:** *Taikicetus inouei* can be identified from closely related taxa (*Diorocetus chichibuensis*, *D. shobarensis*, *Isanacetus laticephalus*, *Parietobalaena* spp.*, Pinocetus polonicus*, *Tiphyocetus temblorensis*) having an anteriorly swollen short zygomatic process (length vs width of the zygomatic process; high triangular coronoid process; and weak angular process, which does not reach as far posterior as the mandibular condyle. *Taikicetus inouei* can be identified by following unique combination of characters; outline of suture between maxillae and palatines forming a posteriorly pointing V-shape (Character 20), convex lateral edge of supraoccipital convex in dorsal view (Character 112), tip of postglenoid process in lateral view pointing ventrally (Character 117), and outline of postglenoid process distinctly wider than high in anterior or posterior view (Character 120).

**Locality:** AMP 35 was found as float from a riverbed of the Rekifune River, Taiki Town, Hokkaido, Japan by Mr. Kiyokazu Inoue in 1991 (Egashira & Kimura, 1998; Kimura, 2004). The site is about 1.5 km southwest from Kyouwa district (Fig. 1): Latitude 42°32′41″N, longitude 143°9′55″E.

**Horizon and age:** There is one basaltic rock layer upstream from the locality, the Green-Tuff Rekifune Formation, which underlies the Middle Miocene Nupinai Group. The Nupinai Group includes Perupune, Hikatagawa and Nupinai Formations (from bottom to top). Pliocene to Recent deposits overly the Nupinai Group (Matsushita et al., 1979). The Perupune Formation consists of green-dark gray conglomerate and sandstone. Hikatagawa Formation consists of sandstone. The Nupinai Formation consists of black to dark gray sandy carbonaceous siltstone. The Pliocene to recent deposits consists of gravel.

AMP 35 was enclosed in a gray block of fine sandstone, which includes black rounded gravels as a float. The source formation of AMP 35 is identified as the Hikatagawa Formation based on its lithology as described by Egashira & Kimura (1998).

There are no direct age estimations for the Nupinai, Hikatagawa and Perupune Formations. The overlying Taiki Group is reportedly in the *Thalassiosira yabei* to *Neodenticula kamtschatica* diatom zone (11.5–5.5 Ma) (Yamaguchi, 1989). Fission track age of the underlying Green-Tuff Rekifune Formation was reported as 14.2 ±1 Ma (Koshimizu & Kim, 1986). Here, we have 15.2–11.5 Ma interval (Langhian to Serravallian; Middle Miocene) as the maximum possible range for the Hikatagawa Formation.

### Description

Bone surfaces of the specimen are eroded in places, and major erosion is noted below. The original position of the AMP 35 elements in the sandstone block was described in Egashira & Kimura (1998). The right mandible and atlas were almost articulated with the skull, and the right tympanic bulla was found on the ventral side of the skull, slightly displaced from its anatomical position.

**Ontogeny:** The periotic is in situ and the long tympanoperiotic posterior process is fused with the skull, indicating that the animal was adult (Geisler & Luo, 1996; Kasuya, 1973). The sutures between the basisphenoid/presphenoid, basioccipital/basisphenoid and exoccipital/supraoccipital on the skull are fused. The sutures between maxilla/premaxilla and squamosal/parietal are closed. Based on these conditions, AMP 35 is identified as an adult individual.

**Figure 2:**
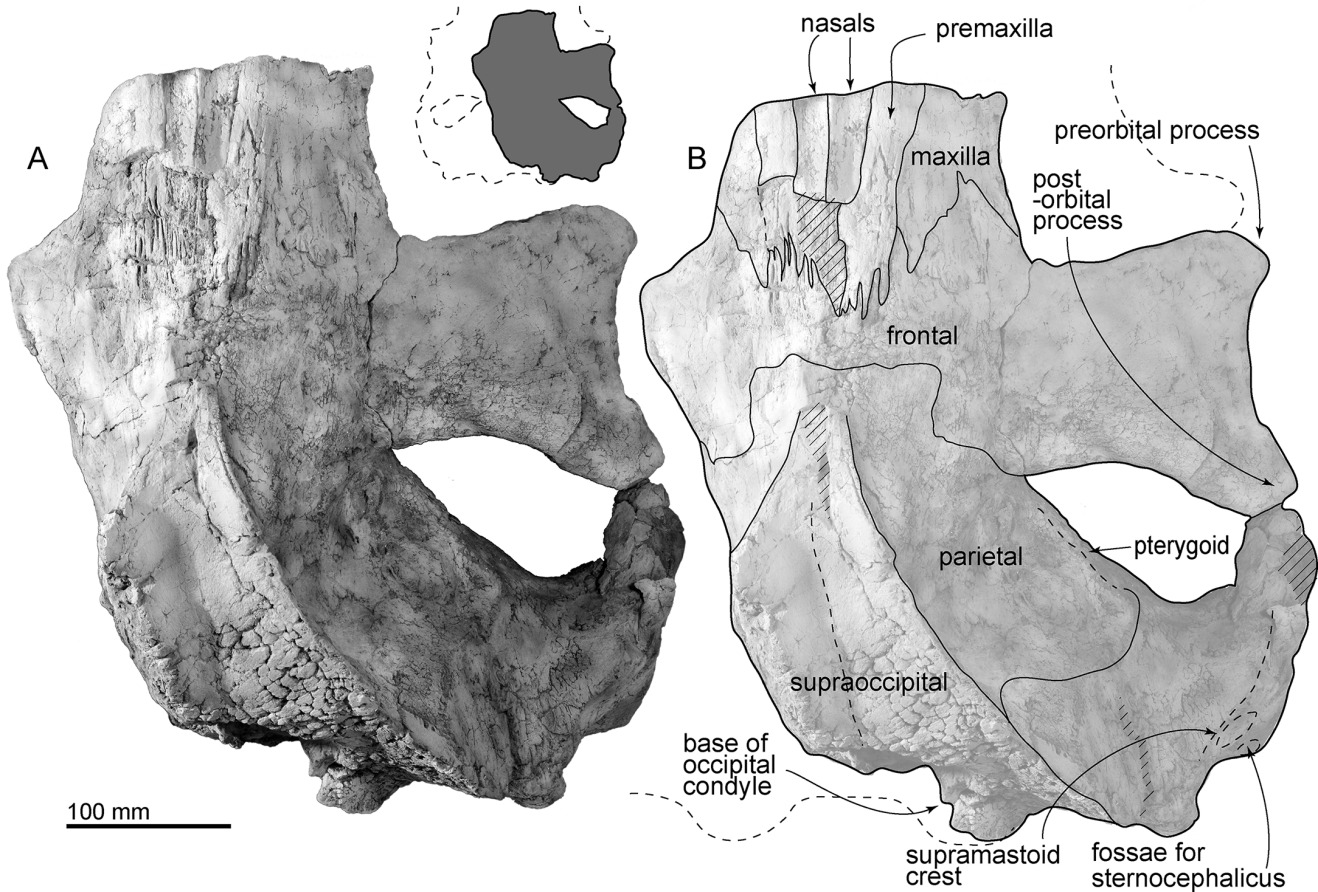
Skull of AMP 35, *Taikicetus inouei* in dorsal view. (A) Photo; (B) line art. Small illustration shows a preserved part.

**Premaxilla:** The right preserved premaxilla is long (122+ mm) and narrow (23 mm wide), and its medial and lateral borders are parallel sided (Fig. 2). The dorsal surface of the premaxilla is weakly convex dorsally. The ascending process of the premaxilla extends posteriorly and contacts the frontal. The premaxilla covers and contacts the maxilla laterally. Medially, the broken section shows that the premaxilla underlies the nasals.

**Maxilla:** The right preserved posterolateral part of the maxilla covers the frontal on the base of the rostrum (Fig. 2). The rostrum base of the maxilla forms a gentle incline which is laterally lower and medially higher (Fig. 5).

**Frontal:** The supraorbital process of the frontal has a ventrolaterally projected thin plate. The anterior border of the frontal is triangular in dorsal view and contacts the maxilla (Fig. 2). The frontals are fused along the median line. The frontal/parietal suture is closed and transversely straight in the middle, and from there extends posteriorly and laterally (Fig. 2). The supraorbital process widens laterally. A robust postorbital process projects posterolaterally, and the posterior border of the frontal is curved. Its posterolateral edge has a weak depression for the zygomatic process of the squamosal. Between the pre- and postorbital processes (135 mm long), the lateral margin of the orbit is excavated as a strong arc in dorsal view. Ventrally, the distinct preorbital and postorbital ridges form a medially pointed triangle. These two ridges are blunt at the lateral part and become medially higher, forming a groove. The medial part of the preorbital ridge is wider than the posterior ridge. The optic canal is oval in cross section and runs medially. The ethmoid foramen and orbitosphenoid are not visible.

**Nasal:** The nasals are anteroposteriorly longer than wide (54 mm preserved long and 18 mm wide), but the anterior border is missing due to damage (Fig. 2). The medial and lateral borders are straight and converge posteriorly gradually. The closed internasal suture rises and forms a very weak anteroposteriorly long crest on the median plane. The posterior part of the nasal is damaged, and it is not clear where it ends.

**Figure 3:**
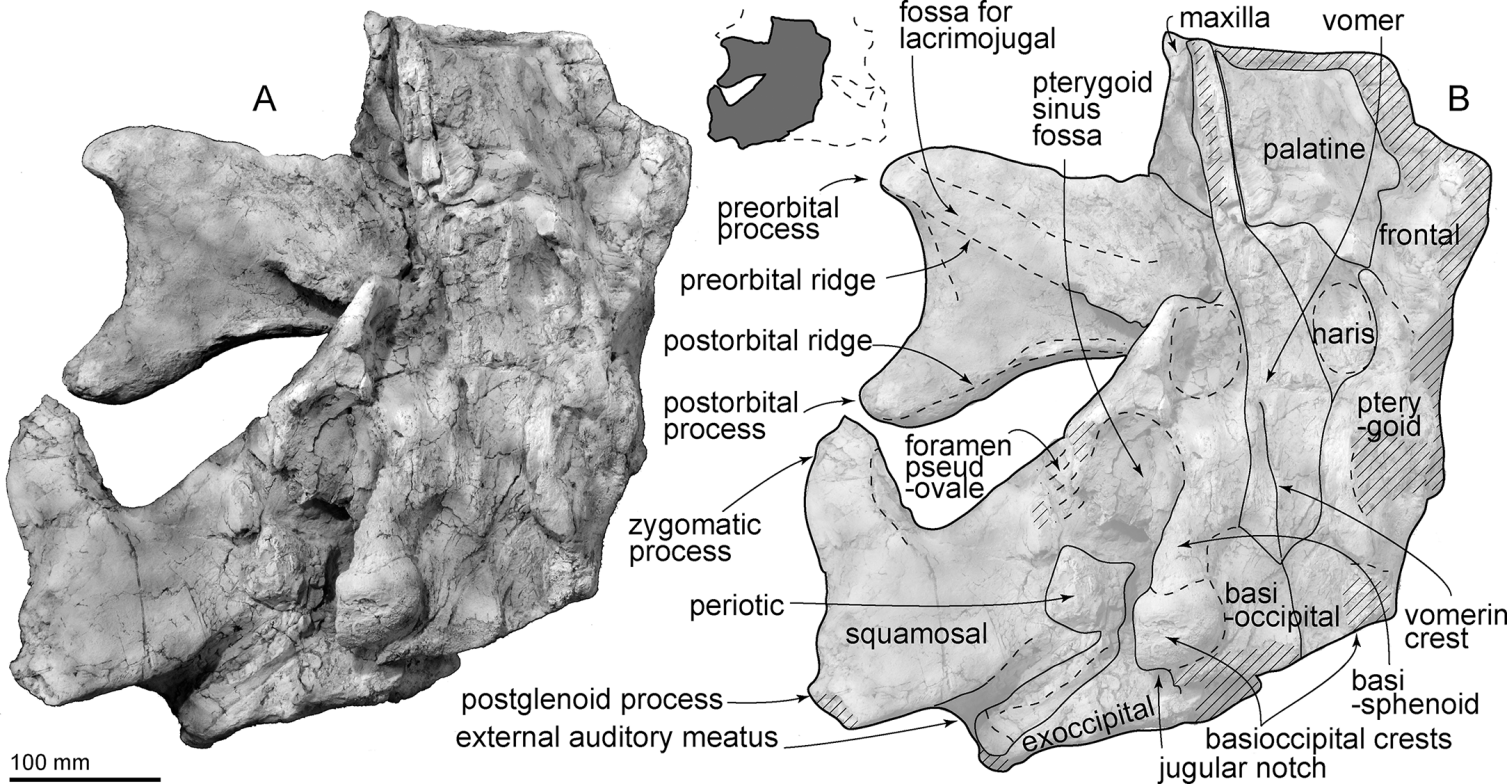
Skull of AMP 35, *Taikicetus inouei* in ventral view. (A) Photo; (B) line art. Small illustration shows a preserved part.

**Figure 4:**
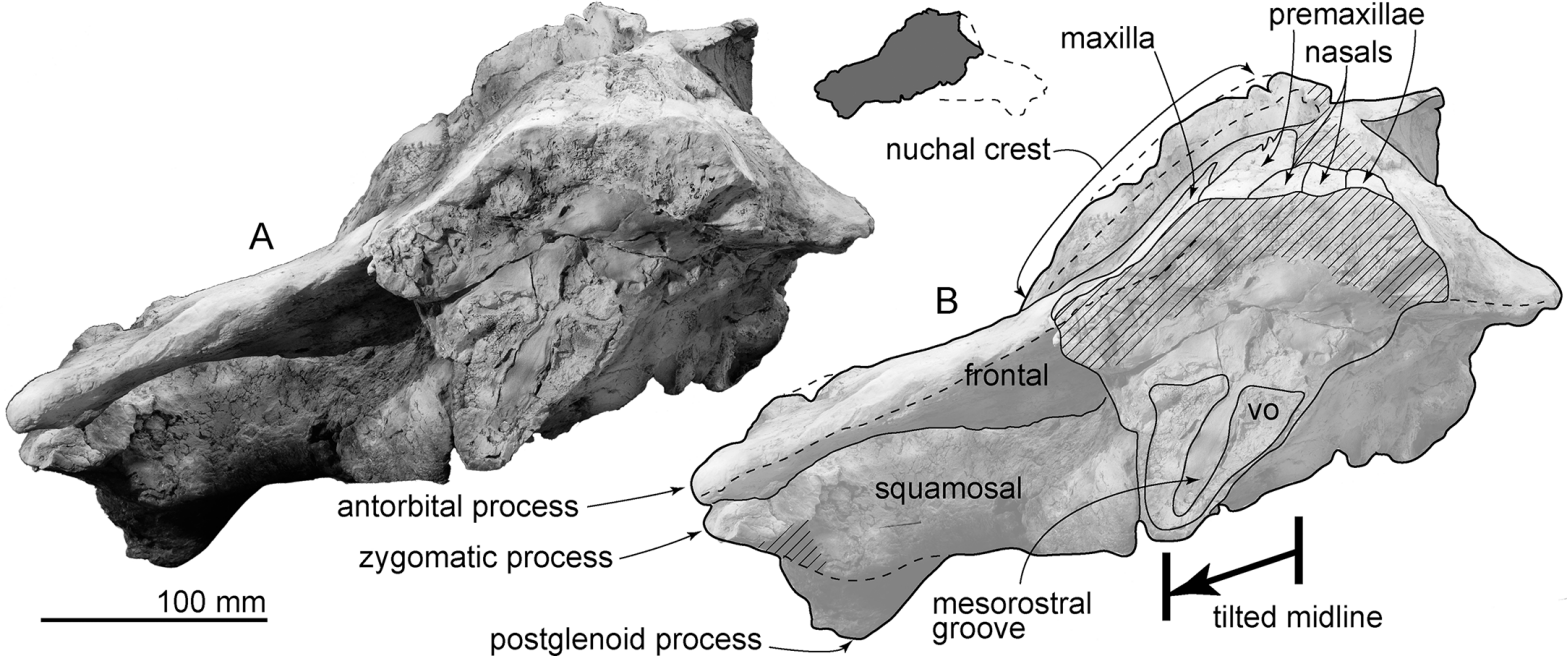
Skull of AMP 35, *Taikicetus inouei* in anterior view. (A) Photo; (B) line art. Small illustration shows a preserved part.

**Vomer:** The skull was deformed after burial, as is evidenced by the clockwise deformation of the skull in anterior view (Fig. 4). Anteriorly, the vomer is dorsoventrally high (70 mm) and wider dorsally (67 mm), than ventrally. The mesorostral canal is dorsoventrally high (60 mm), having an elliptical shape that is open dorsally. Ventrally (Fig. 3), the vomer is exposed between the palatines, and contacts the basisphenoid posteriorly. The ventral surface of the posterior end of the vomer shows a low vomerine crest.

**Figure 5:**
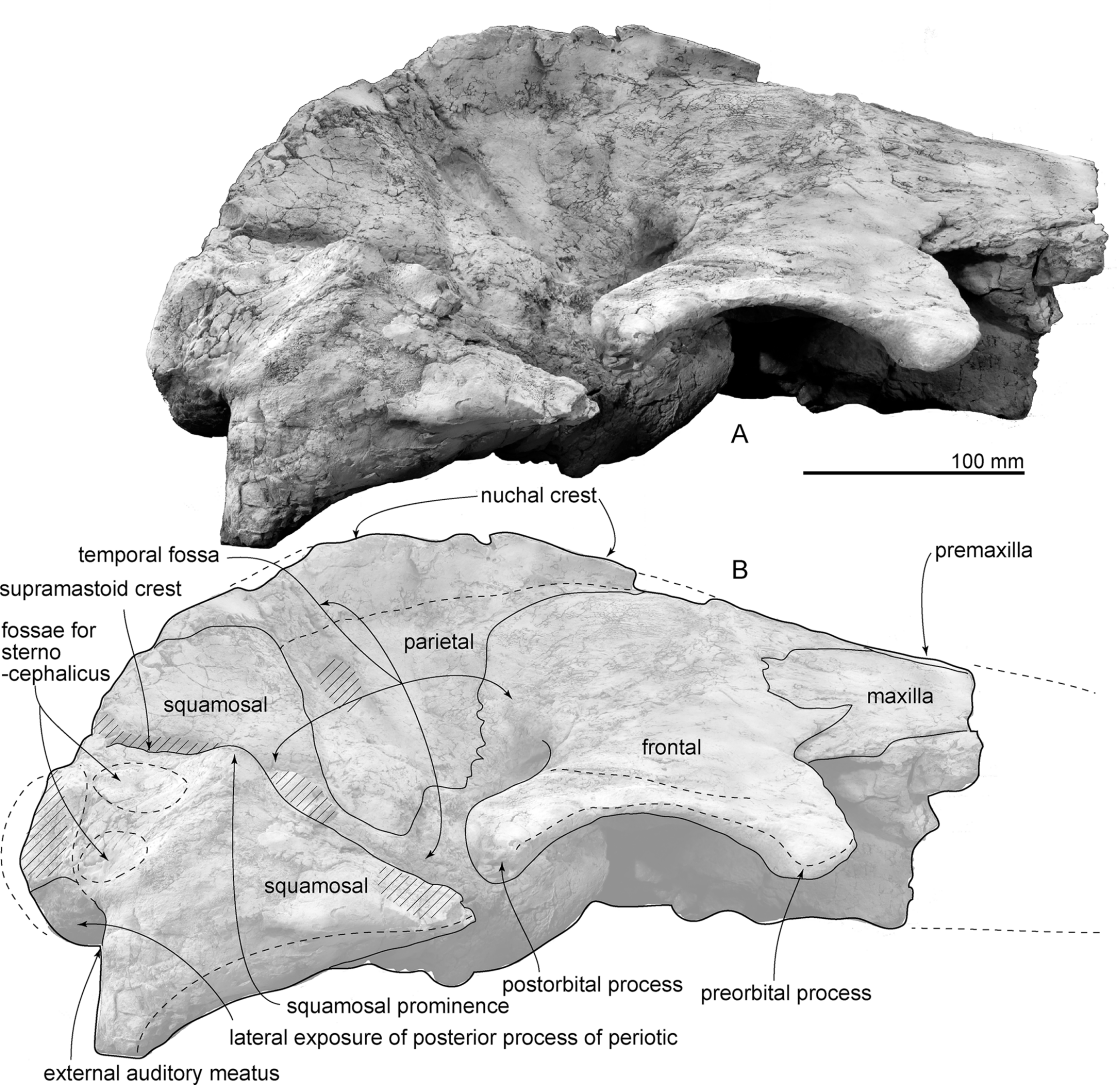
Skull of AMP 35, *Taikicetus inouei* in right lateral view. (A) Photo; (B) line art.

**Palatine:** In ventral view, the palatines cover the vomer laterally (Fig. 3). The posterior end of the palatine is not preserved.

**Parietal:** The parietal is located anterodorsal to the squamosal and posterior to the frontal (Fig. 2). The parietal forms the anteromedial part of a wide temporal fossa. Dorsally, the parietal contributes to the anterior part of the strong nuchal crest. Egashira & Kimura (1998) considered a 4 cm tubercle on the lateral surface of the parietal as an autapomorphy. However, the bone in this area shows fresh broken margins, so the origin of this bone is not clear.

**Pterygoid:** The ventral surface of the pterygoid is damaged (Fig. 3), and forms lateral and posterior parts of the internal bony nares. The anterior pterygoid/palatine and posterior pterygoid/squamosal sutures are unclear. Posteriorly, the pterygoid sinus fossa is elliptical, and is ventromedially covered by the lateral part of the basisphenoid.

**Basioccipital/basisphenoid:** In ventral view (Fig. 3), the anterior part of the basisphenoid is covered by the posterior end of the vomer. At the center of the basioccipital basin, there is a pair of depressions, just posterior to the vomer. The basioccipital/basisphenoid synchondrosis is fused at the center, but is unclear on the basioccipital crest. The basioccipital crest is dorsoventrally thickened. Its lateral margin is trapezoidal in ventral view.

**Supraoccipital:** In dorsal view (Fig. 2), the supraoccipital forms a weakly curved and laterally concave triangular nuchal crest, which is formed with the parietal. On the median plane, the supraoccipital has a longitudinal depression.

**Figure 6:**
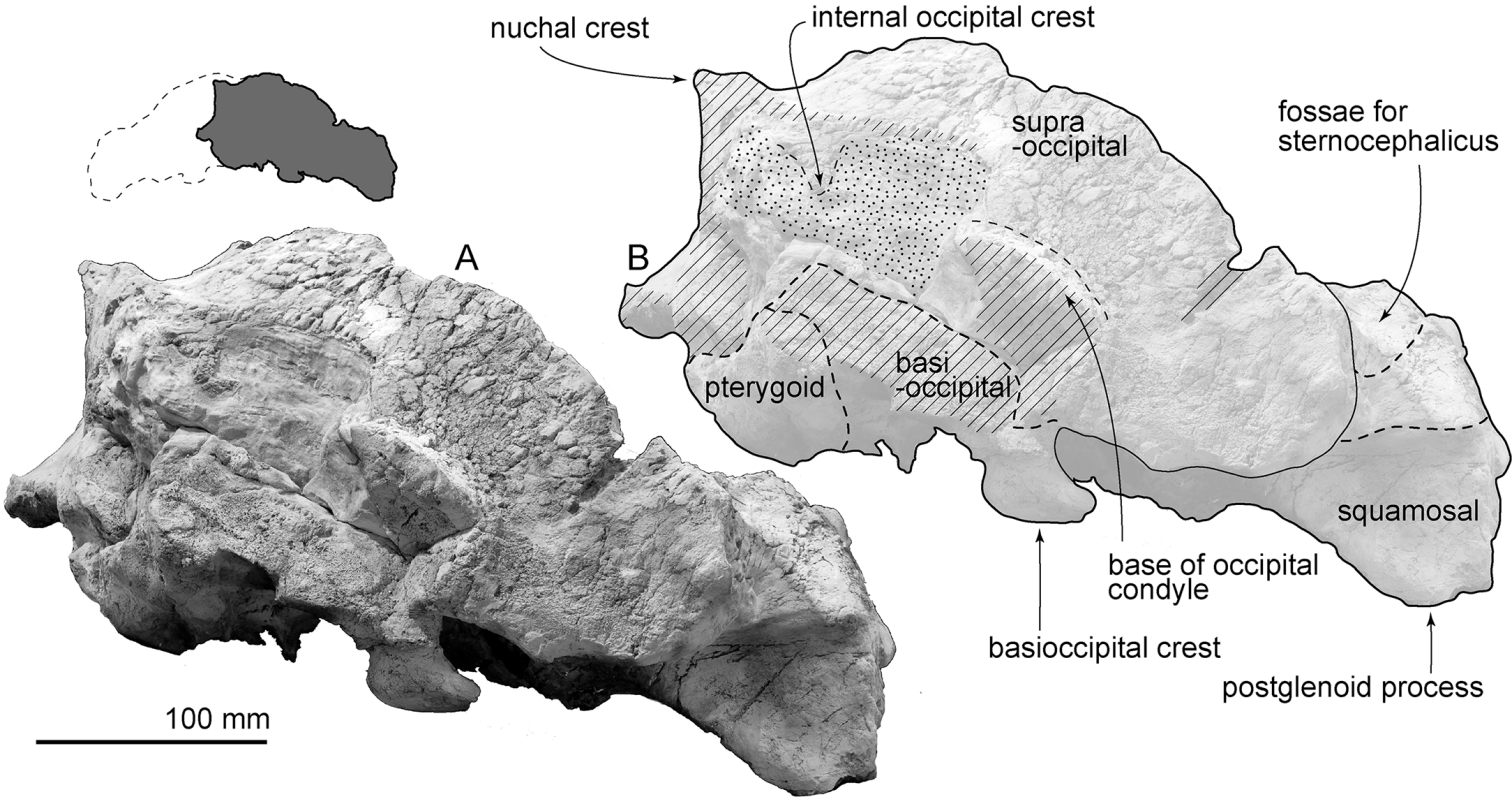
Skull of AMP 35, *Taikicetus inouei* in posterior view. (A) Photo; (B) line art. Small illustration shows a preserved part.

**Figure 7:**
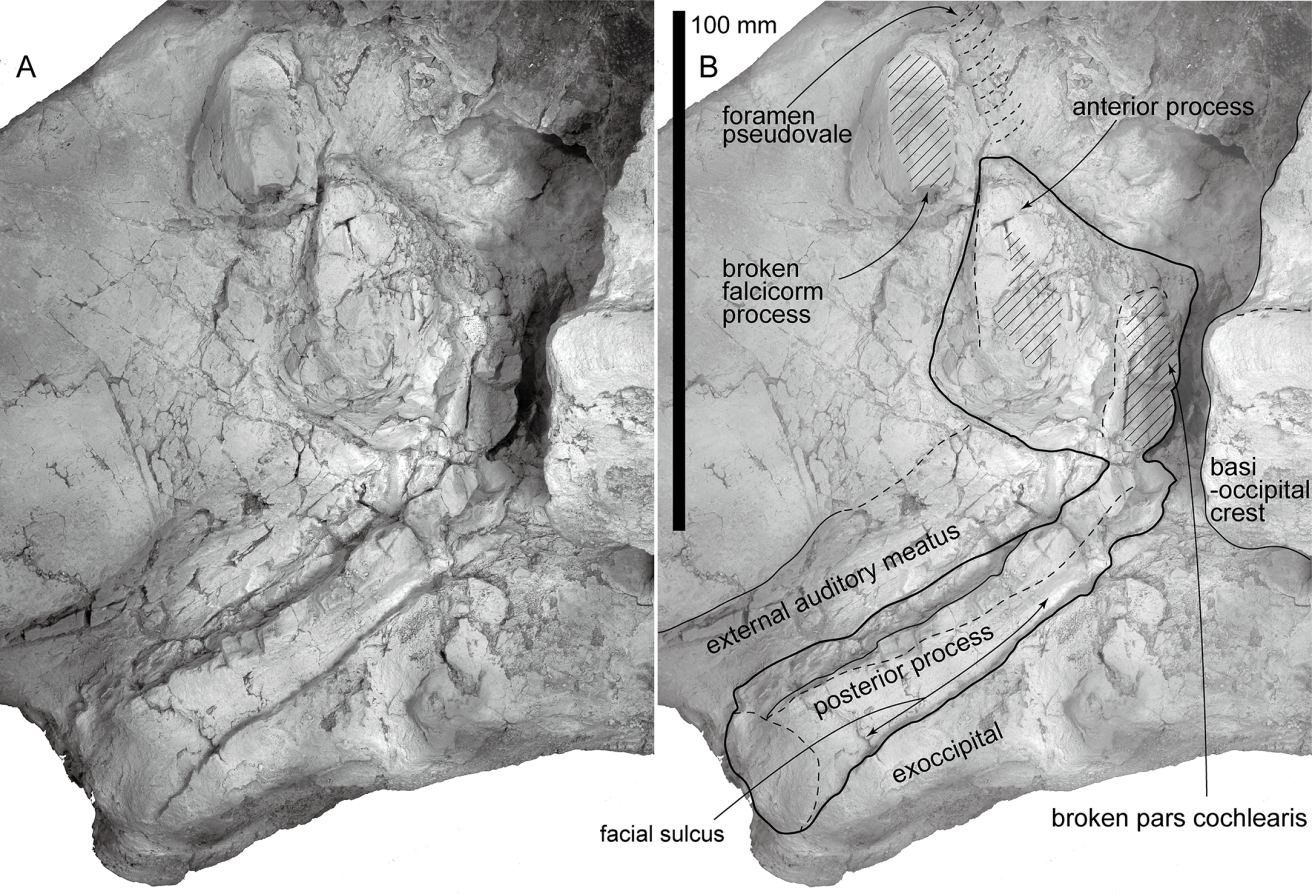
AMP 35, *Taikicetus inouei*, right periotic in situ. (A) Photo; (B) line art.

**Exoccipital:** In ventral view (Fig. 3), the exoccipital is exposed as a plate, which is medially thicker and laterally thinner. It is near the posterior process of the tympanoperiotic (Fig. 7), and bares a shallow jugular notch medially. Posteriorly, the base of the right occipital condyle is broken (Fig. 6).

**Squamosal:** In dorsal view (Fig. 2), the squamosal forms the posterolateral border of the huge triangular temporal fossa. The squamosal has an anteriorly swollen short zygomatic process, which is mediolaterally thick, with a sharp dorsal supramastoid crest. Medial to the supramastoid crest, there is a shallow and wide ventral floor of the temporal fossa. In lateral view, the squamosal is a triangle and is anteroposteriorly slightly longer than its height. There are two fossae for the sternocephalicus on the lateral surface of the squamosal, just anterior to the exoccipital. On the ventral surface of the squamosal a weakly excavated glenoid fossa is posterolaterally restricted by a dorsoventrally low and anteroposteriorly short postglenoid process. The posterior surface of the postglenoid process is nearly vertical. Posterior to the postglenoid process, a transversely long, funnel-shaped external auditory meatus (137 mm from the falciform process to the lateral edge of the postglenoid process) opens laterally and ventrally (40 mm anteroposterior long and 37 mm high at the end of the meatus). The posterior part of the falciform process fits the lateral surface of the anterior process of the periotic. The anteromedial part of the squamosal has a low damaged falciform process anterolateral to the anterior process of the periotic and posterior to the foramen pseudovale. The ventral wall of the foramen pseudovale is broken, posterolateral to the pterygoid sinus fossa.

**Periotic:** The left periotic is in situ and its ventral surface is eroded (Fig. 7). In ventral view, the anterior process is short and triangular. Posteromedial to the anterior process, there is an area of massive bone, which is the broken base of the pars cochlearis. The long compound posterior process of the tympanoperiotic (about 94 mm) projects posterolaterally. The process widens posterolaterally gradually, and has a small rounded lateral exposure (25 mm long). The facial sulcus on the posterior process runs posterolaterally, just anterior to the exoccipital.

**Figure 8:**
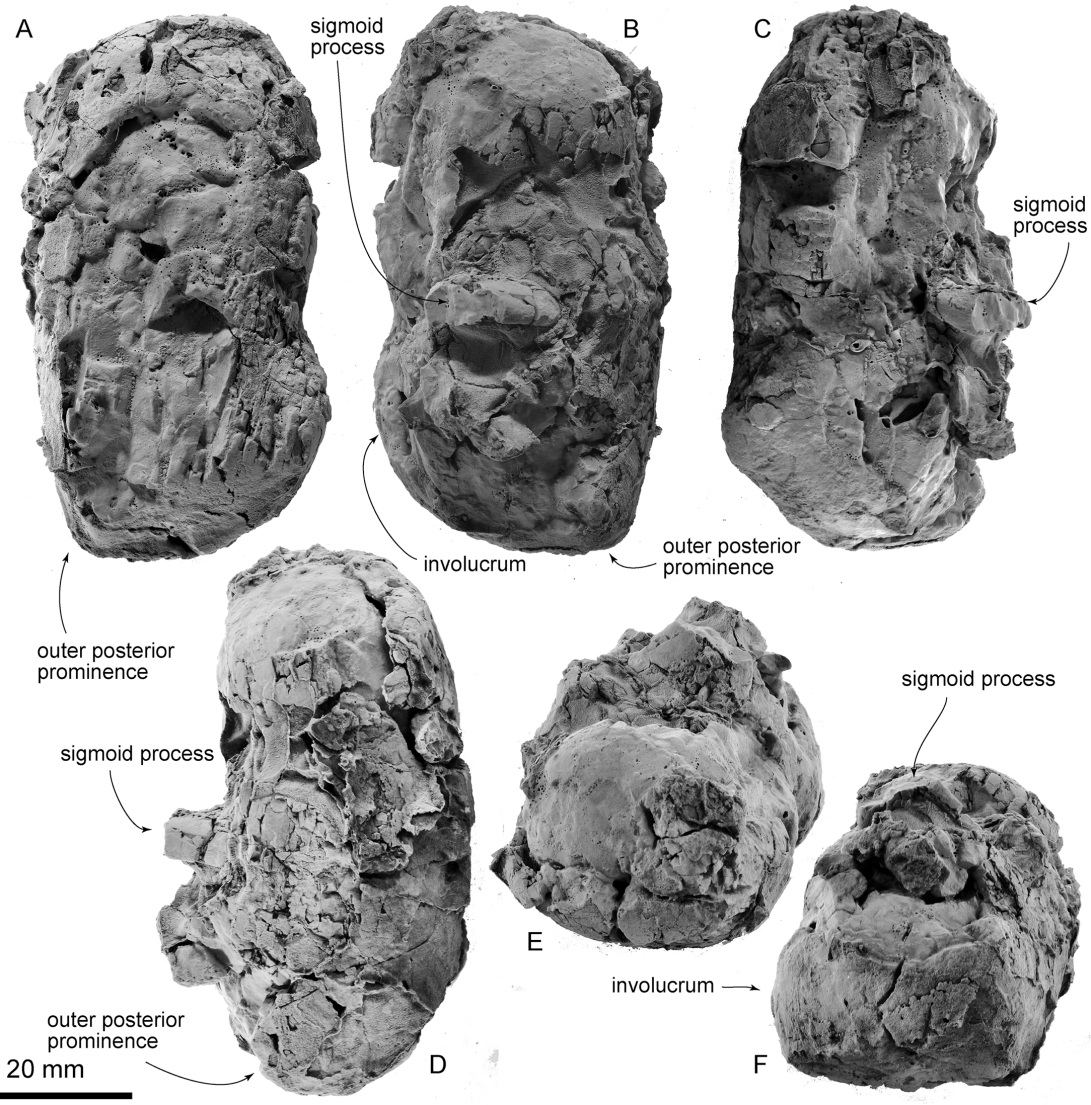
AMP 35, *Taikicetus inouei*, right tympanic bulla. (A) Medial view; (B) lateral view; (C) dorsal view; (D) ventral view; (E) anterior view; (F) posterior view.

**Tympanic bulla:** The tympanic bulla has strongly worn surfaces, and this has obliterated superficial structures (Fig. 8). The outline of the tympanic bulla is a long trapezoid. In medial view, the broken base of the outer lip is preserved. The posterior part of the involucrum is swollen. The outer posterior prominence forms an acute angle. The sigmoid process is located at its mid-length, this process is anteroposteriorly long (6.0 mm) and has a smooth surface. Its lateral edge is broken away.

**Figure 9:**
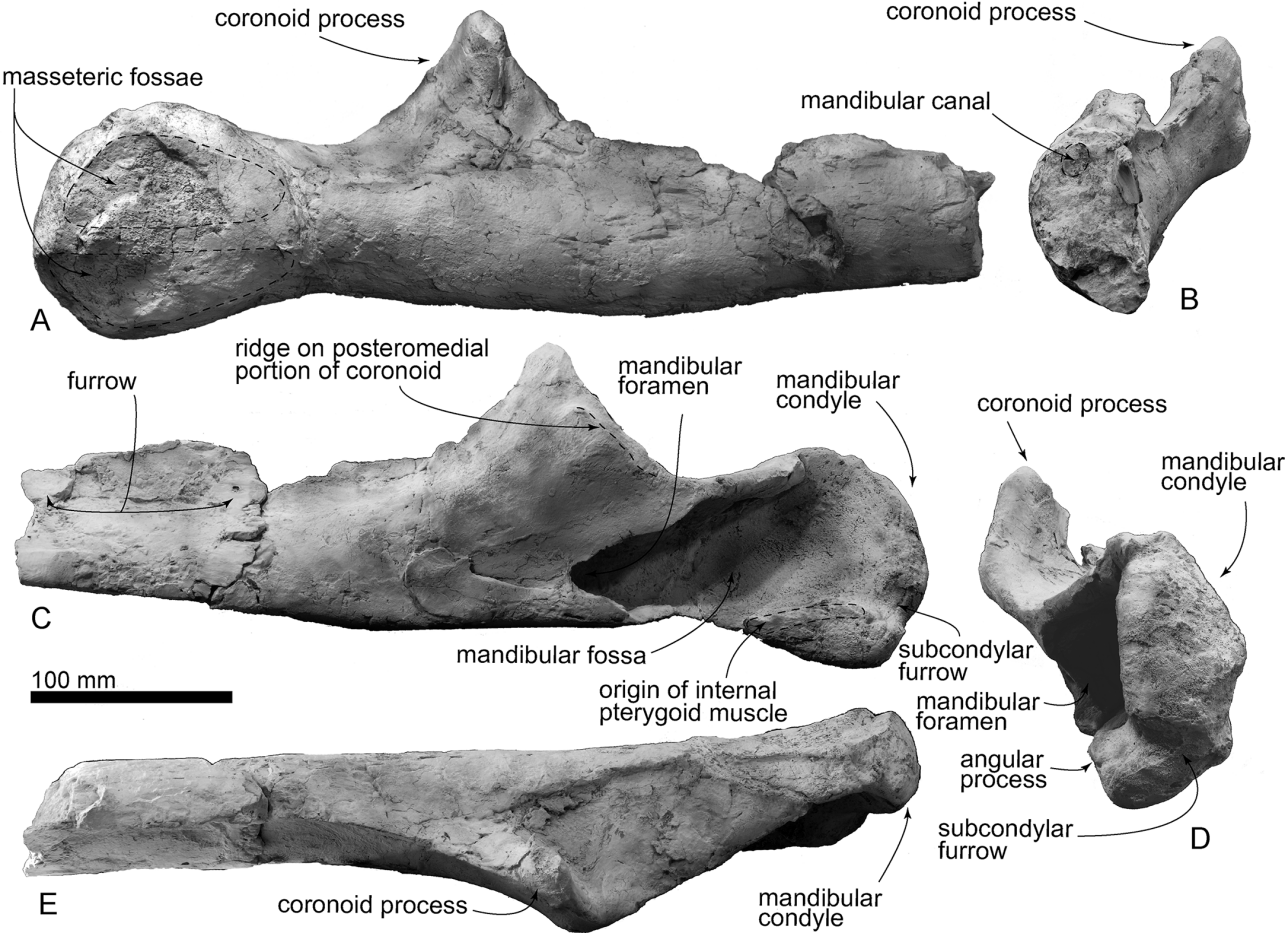
AMP 35, *Taikicetus inouei*, right mandible. (A) Lateral view; (B) anterior view; (C) medial view; (D) posterior view; (E) dorsal view.

**Mandible:** The right mandible preserves the posterior end (Fig. 9). The most anterior preserved cross section has a crescentic shape, with an anteroposteriorly long furrow at the mediodorsal part of the body, and the dorsal border is sharper than the ventral border. At the most anterior preserved cross section, an oval shaped mandibular canal is located dorsally. Anterior to the well-developed coronoid process, the dorsal border is sharp. There is a weak ridge on the posteromedial portion of the coronoid process, which runs close and parallel to the posterior border of the coronoid process. This ridge might be the area of insertion of the temporal muscle, consistent with the interpretation of the muscle attachments by Schulte (1916) and El Adli, Deméré & Boessenecker (2014). The triangular coronoid process narrows dorsally (67 mm high from the mandibular body). The posterior edge of the coronoid process continues to dorsomedial margin of the mandibular fossa. In anteroposterior views, the coronoid process curves laterally and its dorsal end faces posterolaterally, which provides large fossa for the deep masseter muscle. The medial surface of the coronoid process shows a large tubercle (about 25 mm diameter), which might be an area of attachment for the temporal muscle. On the lateral surface of the posterior end of the mandible, there are two masseteric fossae. The dorsal fossa for the deep masseter m. and the ventral for the superficial masseter. Medially, a large and posteriorly deeper mandibular fossa dominants most of the posterior end of the mandible (75 mm high and 155 mm long). The mandibular condyle is dorsally narrow, ventrally wide and posterodorsally swollen. The angular process is weak and is located anterior to the mandibular condyle. The angular process has a flat surface medially. The dorsal part of the flat surface is for the internal pterygoid muscle. Between the mandibular condyle and angular process, there is a shallow subcondylar furrow.

**Figure 10:**
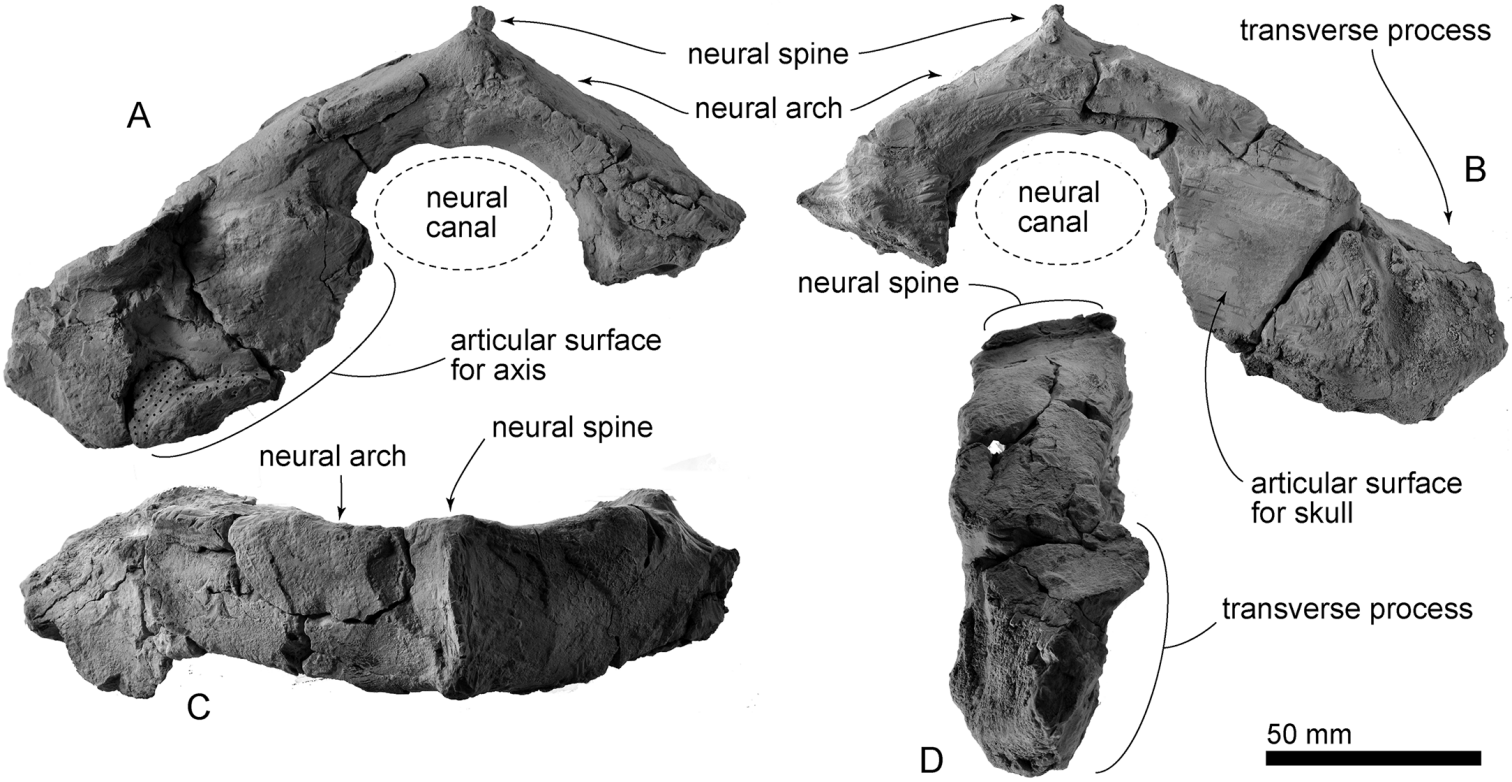
AMP 35, *Taikicetus inouei*, atlas. (A) Posterior view; (B) anterior view; (C) dorsal view; (D) left lateral view.

**Atlas:** The atlas is not fused with the axis (Fig. 10). The atlas is mediolaterally wide (preserved maximum width on the left side is 103 mm) and anteroposteriorly thin (maximum preserved length is 48 mm). The preserved maximum width of the neural canal is 56 mm. The neural spine is low (31 mm high) and has an anteroposteriorly long blade-like ridge (38 mm long). The neural arch is also anteroposteriorly long (38 mm). The articular surface is ventrally broken, but it is high. A laterally damaged transverse process projects posteroventrally, and is slightly tilted posteriorly.

### Phylogenetic analysis

**Figure 11:**
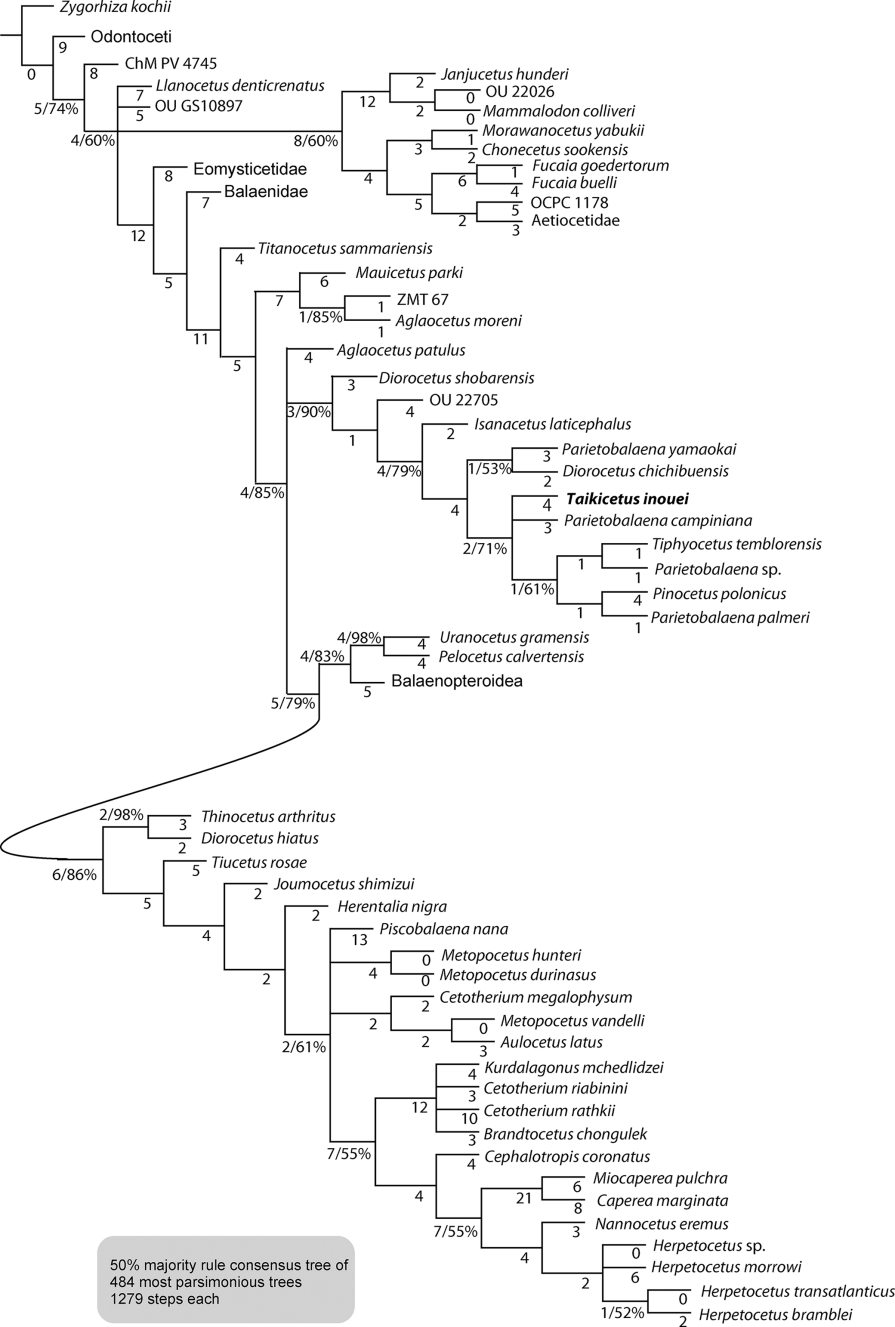
Phylogenetic analysis of *Taikicetus inouei* using only morphological data from the data matrix from Marx, Lambert & de Muizon (2017) with constraint of the molecular tree of Steeman et al. (2009). The clades Aetiocetidae, Eomysticetidae, Balaenidae and Balaenopteroidea, are collapsed. (Cladogram with all taxa shown is in Fig. S1). The branch lengths are labeled. Numbers next to the branch lengths show the percentage of supported shortest trees, but 100% supported nodes are omitted.

The phylogenetic analysis shows 484 shortest trees of 1,279 steps each. The 50% majority rule consensus tree (Fig. 11, top and Fig. S1) shows more or less the same topology of the one of Marx, Lambert & de Muizon (2017). The Balaenidae is placed more basal to *Mauicetus parki* and *Titanocetus sammariensis*. The clade with members of “Cetotheres” sensu lato is nearly the same as in the result in Marx, Lambert & de Muizon (2017) except for the branching pattern of *Aglaocetus patulus* and the addition of *Taikicetus inouei* (AMP 35). Also identical to the result of Marx, Lambert & de Muizon (2017), the Cetotheriidae forms a clade with the Balaenopteroidea, and “a clade comprising *Isanacetus, Parietobalaena* and related taxa” is located basal to the Balaenopteroidea + Cetotheriidae clade.

*Taikicetus inouei* (AMP 35) is placed in “a clade comprising *Isanacetus, Parietobalaena* and related taxa” of Marx, Lambert & de Muizon (2017), which including *D. chichibuensis*, *D. shobarensis*, *I. laticephalus*, OU 22705, *Parietobalaena campiniana, Parietobalaena palmeri, Parietobalaena* sp.*, Parietobalaena yamaokai, Pinocetus polonicus*, and *Tiphyocetus temblorensis.* Previously included species, *A. patulus* is outside of the clade, making an unsolved polytomy with this clade and the (Balaenopteridae + (*Uranocetus* + *Pelocetus*) + Cetotheriidae clade.

## Discussion

The contents of the clade “Cetotheres” sensu lato were *Aglaocetus, Cophocetus, Diorocetus, Isanacetus, Parietobalaena, Pelocetus, Thinocetus* and *Uranocetus* (Marx, Lambert & de Muizon, 2017). “A clade comprising *Isanacetus, Parietobalaena* and related taxa” is formed by members of “Cetotheres” sensu lato including *Taikicetus inouei*. The clade is supported by three synapomorphies. Posteriormost point of the exoccipital in dorsal view locates more anteriorly than the posterior edge of the occipital condyle (character 139, state 0). Relative position of the anterior border of the mandibular foramen in line with the coronoid process (character 227, state 0). Orientation of the transverse processes of the anterior lumbar vertebrae in anterior or posterior view oriented laterally and horizontally (character 245, state 2). Characters 139 and 227 are reversed from 1 to 0, at the node of “a clade comprising *Isanacetus, Parietobalaena* and related taxa” among the Mysticeti phylogeny.

**Comparison to related taxa:** *Taikicetus inouei* can be distinguished from closely related taxa by means of a number of characters. *Taikicetus inouei* has an anteriorly swollen short zygomatic process; the distances from the anterior tip to the lateral end of the temporal crest makes up 80% of the maximum width anterior to the level of the lateral end of the temporal crest. The ratios of the others are about 50%). *Taikicetus inouei* also has a high coronoid process; the distances between the dorsal tip of the coronoid process to the dorsal line of the body of the mandible is 90% of the smallest height of the body of the mandible posterior to the coronoid process. The ratios of the others are 50–70%). *Taikicetus inouei* also has a short angular process, which stops anterior to the mandibular condyle. *D. chichibuensis* Yoshida, Kimura & Hasegawa (2003) and species thought to be related to it (although no phylogenetic analysis was done), *Cophocetus oregonensis* Packard & Kellogg (1934) differ from *Taikicetus inouei* by the latter having an excavated posterior margin of the supraorbital process of the frontal; wider exoccipital; and a non straight posterior border of the maxilla. Compared to *D. shobarensis* Otsuka & Ota (2008), *Taikicetus inouei* differs in having a stronger degree of lateral projection of the postglenoid process. Compared to *I. laticephalus* Kimura & Ozawa (2002), *Taikicetus inouei* is different in having more blunt anterior process of the periotic. Compared to *Parietobalaena campiniana* Bisconti, Lambert & Bosselaers (2013), *Taikicetus inouei* is different in having more laterally concave lateral margin of the orbit in dorsoventral view; longer pedicle of the occipital condyle; and larger anterior process of the periotic. Compared to *Parietobalaena palmeri* Kellogg (1924), *Taikicetus inouei* is different in having nasals that do not contact to the frontals; nasals anterior to the level of the preorbital process of the frontal; robust postorbital process; and anteroposteriorly short temporal fossa in dorsal view. In comparison to *Parietobalaena yamaokai* Otsuka & Ota (2008), *Taikicetus inouei* is different in having an anteriorly pointed triangular supraoccipital (*Parietobalaena yamaokai* has a rounded anterior end of the supraoccipital); and incipient angular process, which stops anterior to the mandibular condyle. In comparison to *Parietobalaena* sp. (SMNH-VeF-62), which was reported by Kimura, Sakamoto & Hasegawa (1998), *Taikicetus inouei* is different in having posteriorly well projected postorbital process; mediolaterally shorter supraorbital process of the frontal; and more weakly projected angular process of the mandible. Compared to *Pinocetus polonicus* Czyzewska & Ryziewicz (1976), *Taikicetus inouei* differs in having a wider and more flat supraoccipital and mediolaterally shallower excavation of the margin of the orbit. Compared to *Hibacetus hirosei* Otsuka & Ota (2008), *Taikicetus inouei* differs in having much narrower basioccipital crests. *H. hirosei* shows wider basioccipital crests. In comparison to *A. patulus* Kellogg (1968), *Taikicetus inouei* is different in having weaker posterior projection of the postglenoid process. *A. patulus* and *Taikicetus inouei* share conditions such as anteriorly pointed supraoccipital, but these shared conditions are not synapomorphies in our analysis.

**Paleobiogeographic patterns:** Knowledge of *Isanacetus, Parietobalaena*, and related taxa in Japan had grown dramatically by 2010 (Egashira & Kimura, 1998; Kimura & Hasegawa, 2010; Otsuka & Ota, 2008; Yoshida, Kimura & Hasegawa, 2003). *D. shobarensis* and *Parietobalaena yamaokai* from Hiroshima are the southern-most records of “Cetotheres” sensu lato in the Western Pacific. By contrast, the new taxon, *Taikicetus inouei* is the northern-most records of “Cetotheres” sensu lato in the Western Pacific and in Japan. It is the record of “Cetotheres” sensu lato from Hokkaido too. As Tanaka, Furusawa & Barnes (in press) reported the northern-most records in Japan of the Herpetocetinae (the Cetotheriidae sensu stricto) from Hokkaido. Hokkaido is a rich area for the discovery of Miocene-Pliocene fossil Mysticeti area, and has provided basic distributional data for not well-known fossil taxa. Compare to the Cetotheriidae, the record of “Cetotheres” sensu lato is difficult to trace evolutionarily. Basal members of “Cetotheres” sensu lato are known from the Pacific (New Zealand and Japan). One of the oldest records of the group is *I. laticephalus* of the Early Miocene. To explain their evolution and paleogeographic distribution, a better understanding of their diversity is needed.

## Conclusion

Here, we described and examined the phylogenetic position of new genus and species *Taikicetus inouei* (AMP 35) from the Hikatagawa Formation, 15.2–11.5 Ma (Langhian to Serravallian; Middle Miocene) in Taiki Town, Hokkaido, Japan. *Taikicetus inouei* is placed in “a clade comprising *Isanacetus, Parietobalaena* and related taxa” comprising most of members of “Cetotheres” sensu lato such as *D. chichibuensis*, *D. shobarensis*, *I. laticephalus*, *Parietobalaena* spp., *Pinocetus polonicus* and *Tiphyocetus temblorensis. Taikicetus inouei* can be distinguished from the members of “Cetotheres” sensu lato in having a robust zygomatic process; high triangular coronoid process; and weak angular process. *Taikicetus inouei* is the only record of a “Cetothere” sensu lato from Hokkaido and the northern-most record in Japan. Specimens from Cetotheriidae sensu stricto have been reported from Numata Town, Hokkaido. *Taikicetus inouei* expands our knowledge on “Cetotheres” sensu lato morphologically and chronogeographically.

## Supplemental Information

10.7717/peerj.4934/supp-1 Supplemental Information 1 Full 50% majority rule consensus tree.

10.7717/peerj.4934/supp-2 Supplemental Information 2 Cladistic matrix in nex format.

10.7717/peerj.4934/supp-3 Supplemental Information 3 Cladistic matrix in tnt format.

10.7717/peerj.4934/supp-4 Supplemental Information 4 Character list.

10.7717/peerj.4934/supp-5 Supplemental Information 5 Tree file.

## Institutional abbreviations

AMP: Ashoro Museum of Paleontology, Hokkaido, Japan
OU: Geology Museum, Dunedin, University of Otago, New Zealand
SMNH: Saitama Museum of Natural History, Saitama, Japan.

## Additional Information and Declarations

### Data Availability

The following information was supplied regarding data availability: The raw data are provided in the Supplemental Files.

### New Species Registration

The following information was supplied regarding the registration of a newly described species: Publication LSID: urn:lsid:zoobank.org:pub:C2190D5F-D3FF-482F-8580-DF8057B2F3AF; Taikicetus: urn:lsid:zoobank.org:act:22CD27F5-B2CC-4981-85BF-3F5143F24E87; Taikicetus inouei: urn:lsid:zoobank.org:act:A40AFF8B-5CEC-41B9-92D9-B2E13505CA95.
