# Supplementary figures and images for: A new species of Middle Miocene baleen whale from the Nupinai Group, Hikatagawa Formation of Hokkaido, Japan
Source: PeerJ. 2018 Jun 26;6:e4934. doi: 10.7717/peerj.4934 (PMC6025157; doi:10.7717/peerj.4934)

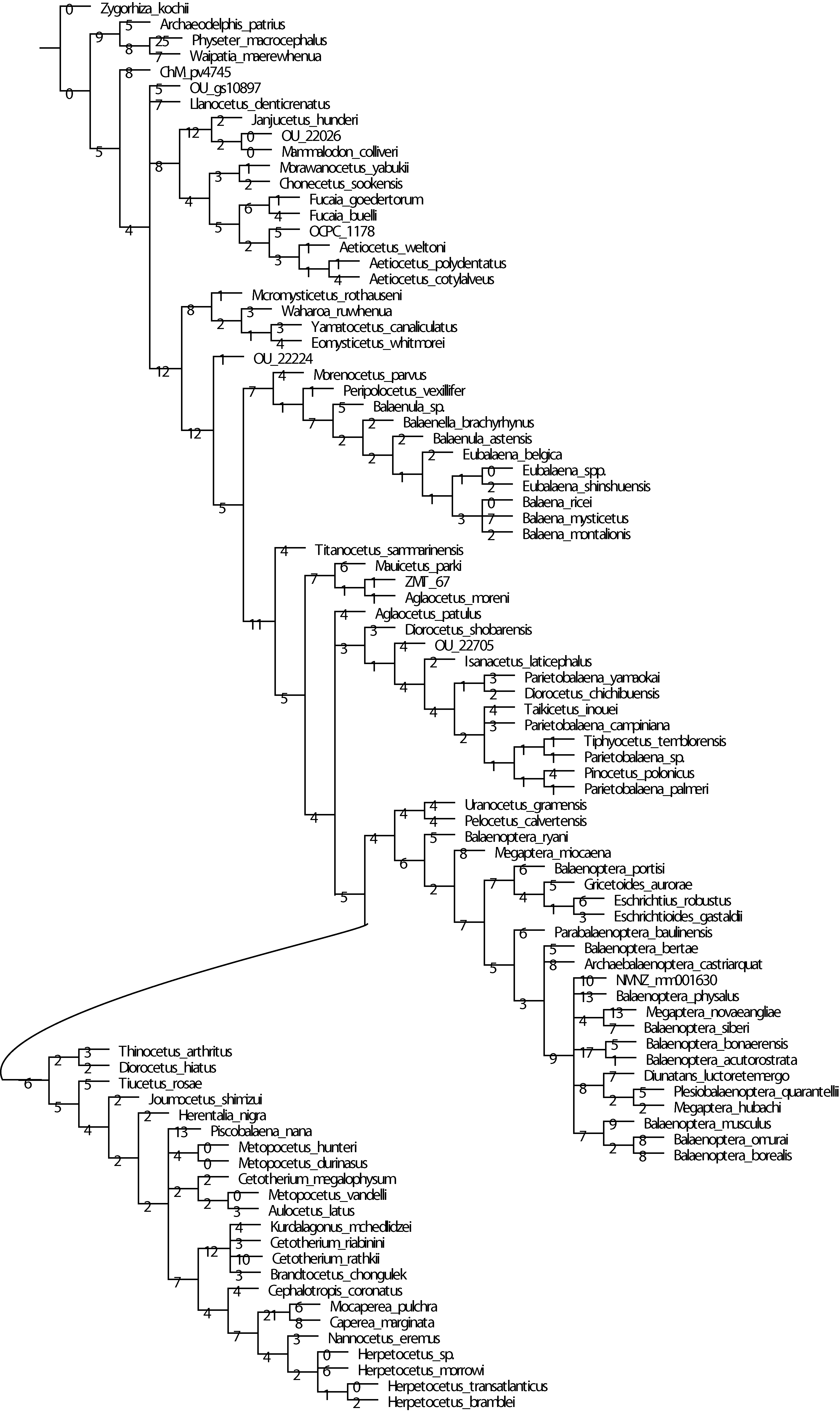

Supplement: Supplemental Information 1 [file peerj-06-4934-s001.png]
